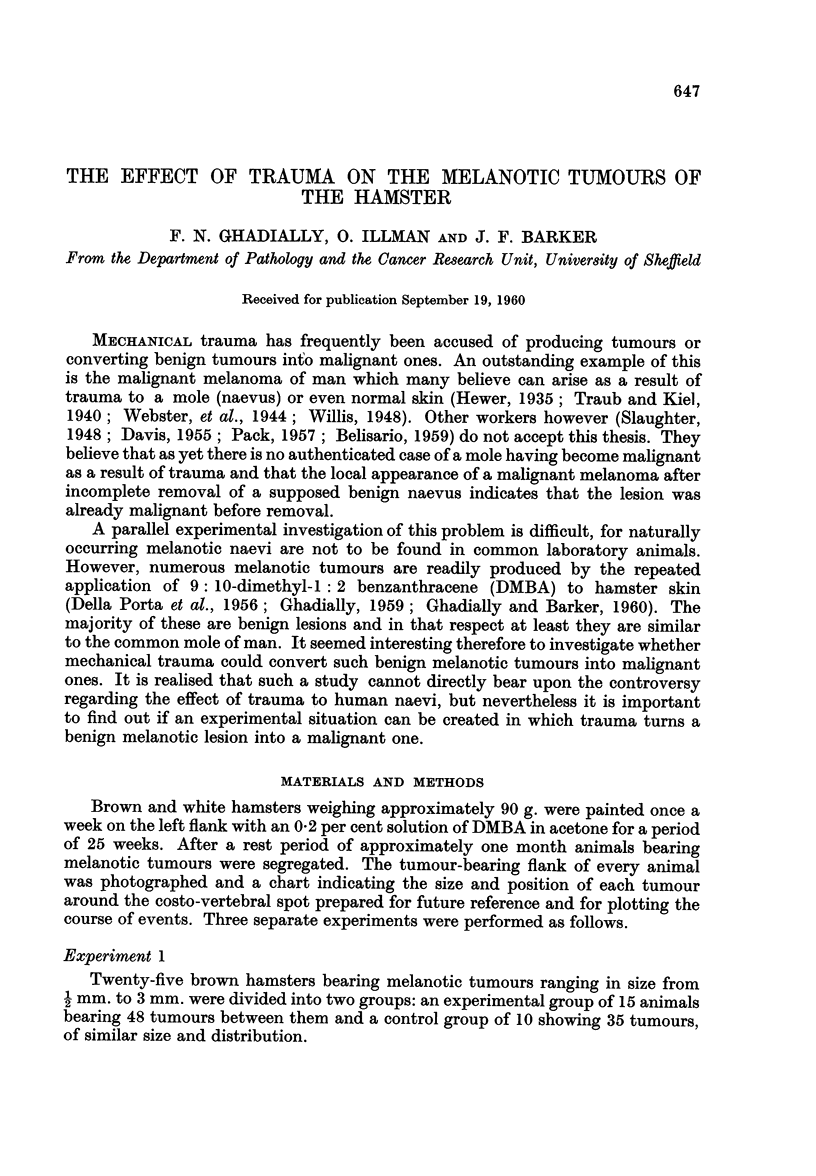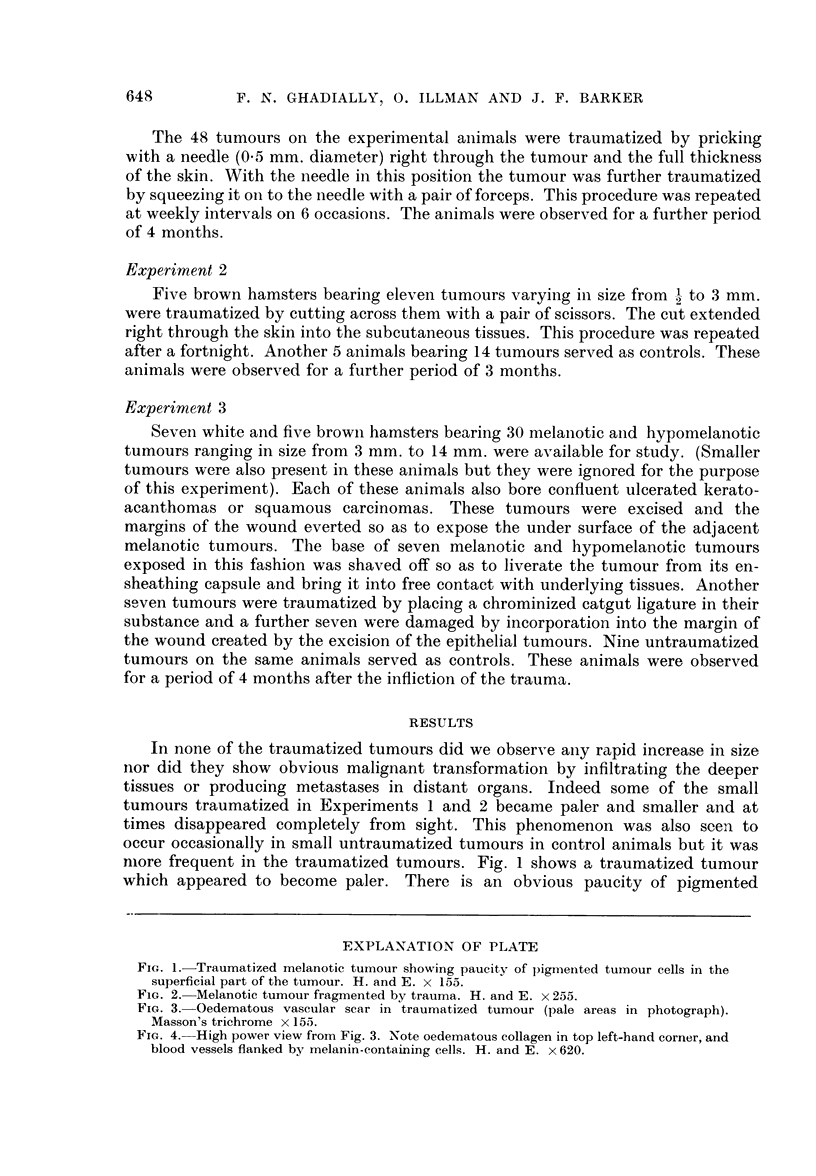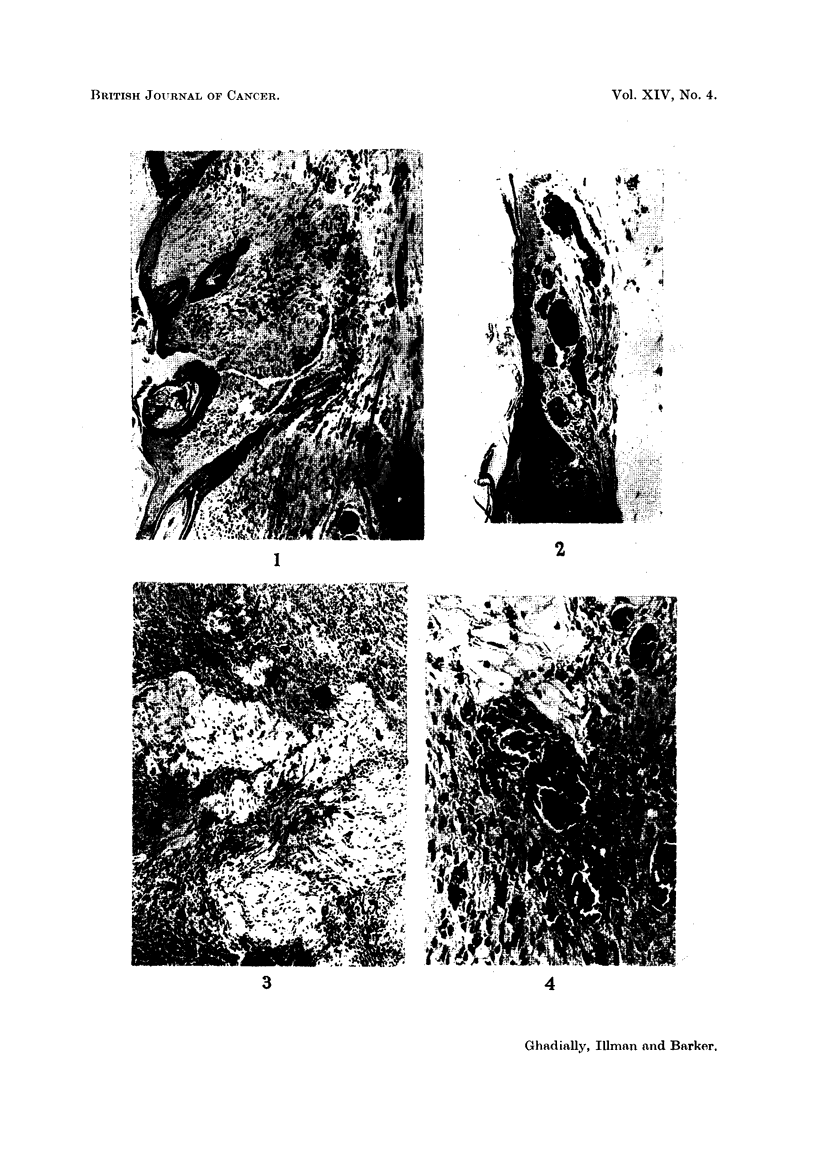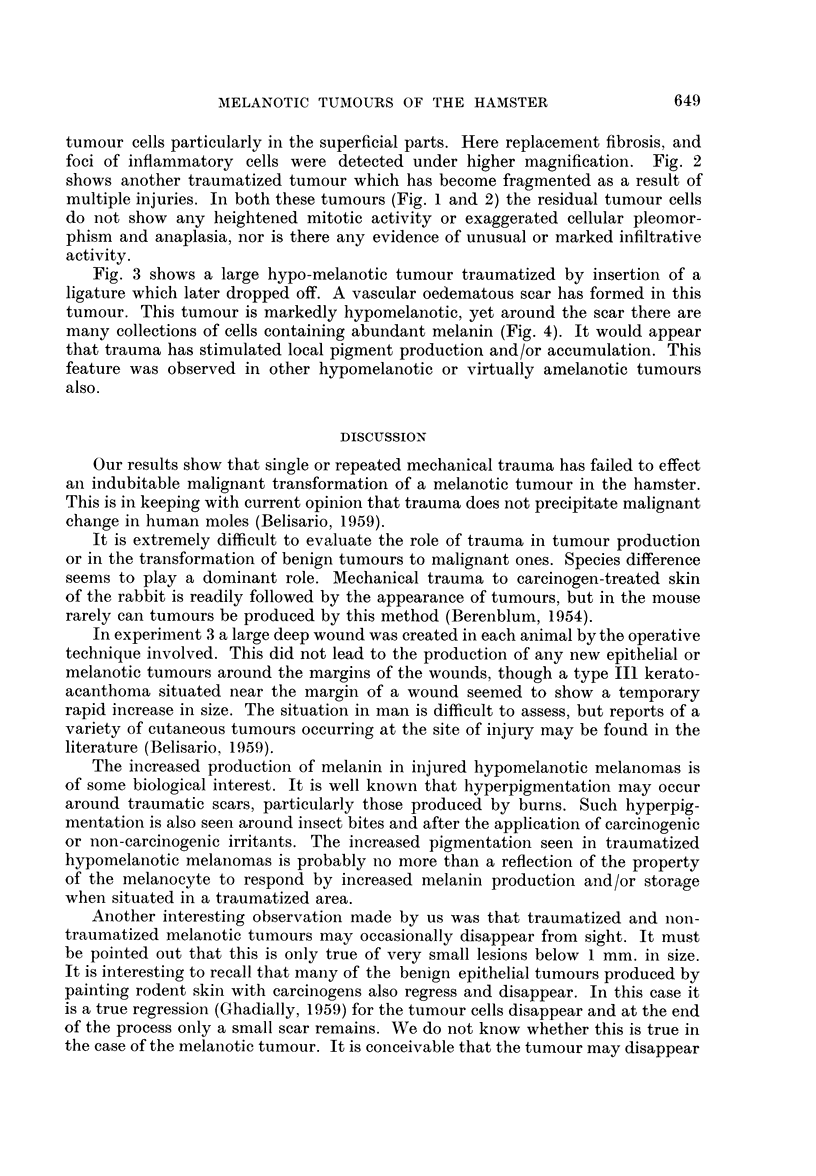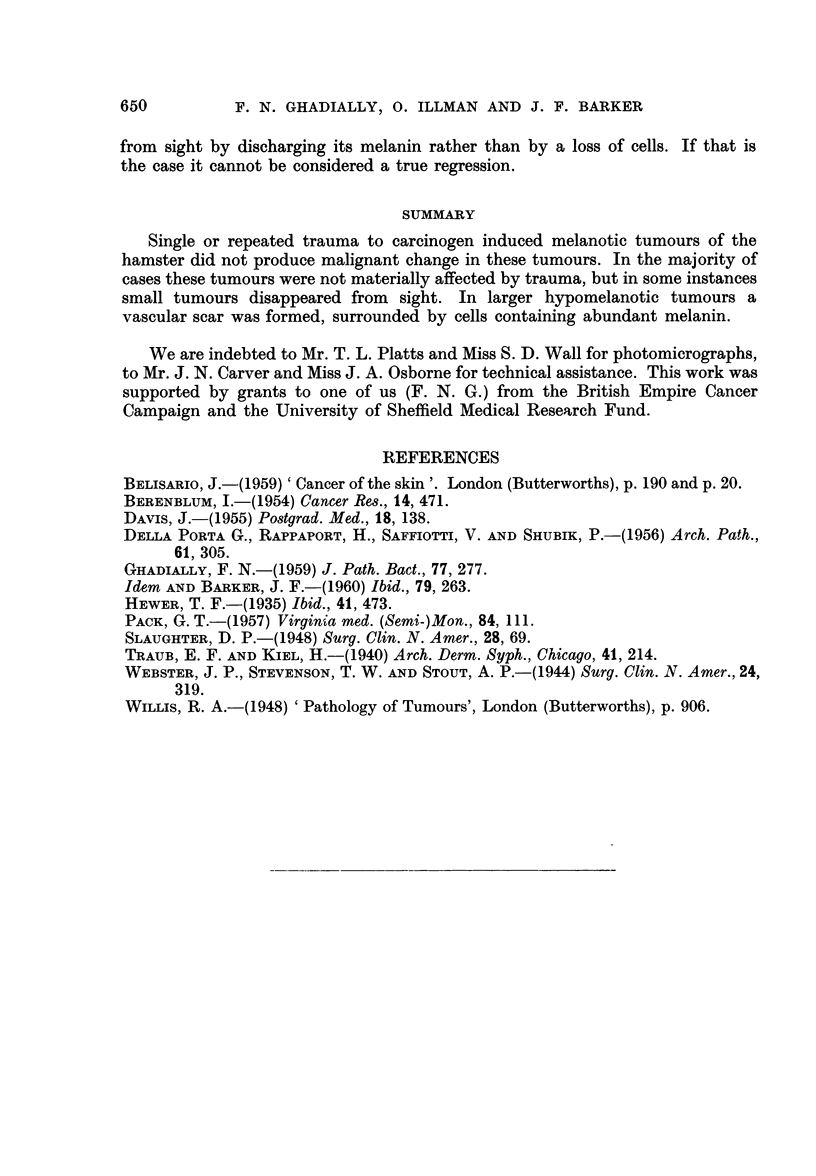# The Effect of Trauma on the Melanotic Tumours of the Hamster

**DOI:** 10.1038/bjc.1960.71

**Published:** 1960-12

**Authors:** F. N. Ghadially, O. Illman, J. F. Barker

## Abstract

**Images:**


### THE EFFECT OF TRAUMA ON THE MELANOTIC TUMOURS OF THE HAMSTER

F. N. GHADIALLY, O. ILLMAN AND J. F. BARKER

*From the Department of Pathology and the Cancer Research Unit, University of Sheffield*

Received for publication September 19, 1960

MECHANICAL trauma has frequently been accused of producing tumours or converting benign tumours into malignant ones. An outstanding example of this is the malignant melanoma of man which many believe can arise as a result of trauma to a mole (naevus) or even normal skin (Hewer, 1935 ; Traub and Kiel, 1940 ; Webster, *et al.*, 1944 ; Willis, 1948). Other workers however (Slaughter, 1948 ; Davis, 1955 ; Pack, 1957 ; Belisario, 1959) do not accept this thesis. They believe that as yet there is no authenticated case of a mole having become malignant as a result of trauma and that the local appearance of a malignant melanoma after incomplete removal of a supposed benign naevus indicates that the lesion was already malignant before removal.

A parallel experimental investigation of this problem is difficult, for naturally occurring melanotic naevi are not to be found in common laboratory animals. However, numerous melanotic tumours are readily produced by the repeated application of 9 : 10-dimethyl-1 : 2 benzanthracene (DMBA) to hamster skin (Della Porta *et al.*, 1956 ; Ghadially, 1959 ; Ghadially and Barker, 1960). The majority of these are benign lesions and in that respect at least they are similar to the common mole of man. It seemed interesting therefore to investigate whether mechanical trauma could convert such benign melanotic tumours into malignant ones. It is realised that such a study cannot directly bear upon the controversy regarding the effect of trauma to human naevi, but nevertheless it is important to find out if an experimental situation can be created in which trauma turns a benign melanotic lesion into a malignant one.

### MATERIALS AND METHODS

Brown and white hamsters weighing approximately 90 g. were painted once a week on the left flank with an 0·2 per cent solution of DMBA in acetone for a period of 25 weeks. After a rest period of approximately one month animals bearing melanotic tumours were segregated. The tumour-bearing flank of every animal was photographed and a chart indicating the size and position of each tumour around the costo-vertebral spot prepared for future reference and for plotting the course of events. Three separate experiments were performed as follows.

### *Experiment* 1

Twenty-five brown hamsters bearing melanotic tumours ranging in size from ½ mm. to 3 mm. were divided into two groups: an experimental group of 15 animals bearing 48 tumours between them and a control group of 10 showing 35 tumours, of similar size and distribution.

The 48 tumours on the experimental animals were traumatized by pricking with a needle (0·5 mm. diameter) right through the tumour and the full thickness of the skin. With the needle in this position the tumour was further traumatized by squeezing it on to the needle with a pair of forceps. This procedure was repeated at weekly intervals on 6 occasions. The animals were observed for a further period of 4 months.

*Experiment* 2

Five brown hamsters bearing eleven tumours varying in size from $\frac{1}{2}$ to 3 mm. were traumatized by cutting across them with a pair of scissors. The cut extended right through the skin into the subcutaneous tissues. This procedure was repeated after a fortnight. Another 5 animals bearing 14 tumours served as controls. These animals were observed for a further period of 3 months.

*Experiment* 3

Seven white and five brown hamsters bearing 30 melanotic and hypomelanotic tumours ranging in size from 3 mm. to 14 mm. were available for study. (Smaller tumours were also present in these animals but they were ignored for the purpose of this experiment). Each of these animals also bore confluent ulcerated kerato-acanthomas or squamous carcinomas. These tumours were excised and the margins of the wound everted so as to expose the under surface of the adjacent melanotic tumours. The base of seven melanotic and hypomelanotic tumours exposed in this fashion was shaved off so as to liverate the tumour from its ensheathing capsule and bring it into free contact with underlying tissues. Another seven tumours were traumatized by placing a chrominized catgut ligature in their substance and a further seven were damaged by incorporation into the margin of the wound created by the excision of the epithelial tumours. Nine untraumatized tumours on the same animals served as controls. These animals were observed for a period of 4 months after the infliction of the trauma.

### RESULTS

In none of the traumatized tumours did we observe any rapid increase in size nor did they show obvious malignant transformation by infiltrating the deeper tissues or producing metastases in distant organs. Indeed some of the small tumours traumatized in Experiments 1 and 2 became paler and smaller and at times disappeared completely from sight. This phenomenon was also seen to occur occasionally in small untraumatized tumours in control animals but it was more frequent in the traumatized tumours. Fig. 1 shows a traumatized tumour which appeared to become paler. There is an obvious paucity of pigmented

---

### EXPLANATION OF PLATE

FIG. 1.—Traumatized melanotic tumour showing paucity of pigmented tumour cells in the superficial part of the tumour. H. and E. × 155.

FIG. 2.—Melanotic tumour fragmented by trauma. H. and E. ×255.

FIG. 3.—Oedematous vascular scar in traumatized tumour (pale areas in photograph). Masson's trichrome ×155.

FIG. 4.—High power view from Fig. 3. Note oedematous collagen in top left-hand corner, and blood vessels flanked by melanin-containing cells. H. and E. ×620.

Ghadially, Illman and Barker.

tumour cells particularly in the superficial parts. Here replacement fibrosis, and foci of inflammatory cells were detected under higher magnification. Fig. 2 shows another traumatized tumour which has become fragmented as a result of multiple injuries. In both these tumours (Fig. 1 and 2) the residual tumour cells do not show any heightened mitotic activity or exaggerated cellular pleomorphism and anaplasia, nor is there any evidence of unusual or marked infiltrative activity.

Fig. 3 shows a large hypo-melanotic tumour traumatized by insertion of a ligature which later dropped off. A vascular oedematous scar has formed in this tumour. This tumour is markedly hypomelanotic, yet around the scar there are many collections of cells containing abundant melanin (Fig. 4). It would appear that trauma has stimulated local pigment production and/or accumulation. This feature was observed in other hypomelanotic or virtually amelanotic tumours also.

### DISCUSSION

Our results show that single or repeated mechanical trauma has failed to effect an indubitable malignant transformation of a melanotic tumour in the hamster. This is in keeping with current opinion that trauma does not precipitate malignant change in human moles (Belisario, 1959).

It is extremely difficult to evaluate the role of trauma in tumour production or in the transformation of benign tumours to malignant ones. Species difference seems to play a dominant role. Mechanical trauma to carcinogen-treated skin of the rabbit is readily followed by the appearance of tumours, but in the mouse rarely can tumours be produced by this method (Berenblum, 1954).

In experiment 3 a large deep wound was created in each animal by the operative technique involved. This did not lead to the production of any new epithelial or melanotic tumours around the margins of the wounds, though a type III kerato-acanthoma situated near the margin of a wound seemed to show a temporary rapid increase in size. The situation in man is difficult to assess, but reports of a variety of cutaneous tumours occurring at the site of injury may be found in the literature (Belisario, 1959).

The increased production of melanin in injured hypomelanotic melanomas is of some biological interest. It is well known that hyperpigmentation may occur around traumatic scars, particularly those produced by burns. Such hyperpigmentation is also seen around insect bites and after the application of carcinogenic or non-carcinogenic irritants. The increased pigmentation seen in traumatized hypomelanotic melanomas is probably no more than a reflection of the property of the melanocyte to respond by increased melanin production and/or storage when situated in a traumatized area.

Another interesting observation made by us was that traumatized and non-traumatized melanotic tumours may occasionally disappear from sight. It must be pointed out that this is only true of very small lesions below 1 mm. in size. It is interesting to recall that many of the benign epithelial tumours produced by painting rodent skin with carcinogens also regress and disappear. In this case it is a true regression (Ghadially, 1959) for the tumour cells disappear and at the end of the process only a small scar remains. We do not know whether this is true in the case of the melanotic tumour. It is conceivable that the tumour may disappear from sight by discharging its melanin rather than by a loss of cells. If that is the case it cannot be considered a true regression.

### SUMMARY

Single or repeated trauma to carcinogen induced melanotic tumours of the hamster did not produce malignant change in these tumours. In the majority of cases these tumours were not materially affected by trauma, but in some instances small tumours disappeared from sight. In larger hypomelanotic tumours a vascular scar was formed, surrounded by cells containing abundant melanin.

We are indebted to Mr. T. L. Platts and Miss S. D. Wall for photomicrographs, to Mr. J. N. Carver and Miss J. A. Osborne for technical assistance. This work was supported by grants to one of us (F. N. G.) from the British Empire Cancer Campaign and the University of Sheffield Medical Research Fund.

### REFERENCES

Belisario, J.—(1959) ' Cancer of the skin '. London (Butterworths), p. 190 and p. 20.
Berenblum, I.—(1954) *Cancer Res.*, **14**, 471.
Davis, J.—(1955) *Postgrad. Med.*, **18**, 138.
Della Porta G., Rappaport, H., Saffiotti, V. and Shubik, P.—(1956) *Arch. Path.*, **61**, 305.
Ghadially, F. N.—(1959) *J. Path. Bact.*, **77**, 277.
*Idem* and Barker, J. F.—(1960) *Ibid.*, **79**, 263.
Hewer, T. F.—(1935) *Ibid.*, **41**, 473.
Pack, G. T.—(1957) *Virginia med. (Semi-)Mon.*, **84**, 111.
Slaughter, D. P.—(1948) *Surg. Clin. N. Amer.*, **28**, 69.
Traub, E. F. and Kiel, H.—(1940) *Arch. Derm. Syph., Chicago*, **41**, 214.
Webster, J. P., Stevenson, T. W. and Stout, A. P.—(1944) *Surg. Clin. N. Amer.*, **24**, 319.
Willis, R. A.—(1948) ' Pathology of Tumours', London (Butterworths), p. 906.